# Mesophilic versus thermophilic anaerobic digestion of cattle manure: methane productivity and microbial ecology

**DOI:** 10.1111/1751-7915.12271

**Published:** 2015-03-04

**Authors:** Veronica Moset, Morten Poulsen, Radziah Wahid, Ole Højberg, Henrik Bjarne Møller

**Affiliations:** 1 Department of Engineering, Aarhus UniversityBlichers Allé 20, DK 8830, Tjele, Denmark; 2 Department of Animal Science, Aarhus UniversityBlichers Allé 20, DK 8830, Tjele, Denmark; 3 Faculty of Chemical Engineering, Universiti Teknologi Mara40450, Shah Alam, Malaysia

## Abstract

In this study, productivity and physicochemical and microbiological (454 sequencing) parameters, as well as environmental criteria, were investigated in anaerobic reactors to contribute to the ongoing debate about the optimal temperature range for treating animal manure, and expand the general knowledge on the relation between microbiological and physicochemical process indicators. For this purpose, two reactor sizes were used (10 m^3^ and 16 l), in which two temperature conditions (35°C and 50°C) were tested. In addition, the effect of the hydraulic retention time was evaluated (16 versus 20 days).

Thermophilic anaerobic digestion showed higher organic matter degradation (especially fiber), higher pH and higher methane (CH_4_) yield, as well as better percentage of ultimate CH_4_ yield retrieved and lower residual CH_4_ emission, when compared with mesophilic conditions. In addition, lower microbial diversity was found in the thermophilic reactors, especially for *Bacteria*, where a clear intensification towards Clostridia class members was evident.

Independent of temperature, some similarities were found in digestates when comparing with animal manure, including low volatile fatty acids concentrations and a high fraction of Euryarchaeota in the total microbial community, in which members of Methanosarcinales dominated for both temperature conditions; these indicators could be considered a sign of process stability.

## Introduction

Anaerobic digestion of organic by-products is a key technology for producing high-value bioenergy in form of biogas. However, anaerobic digestion is a highly complex process which nowadays is still the focus of multiple investigations, being the microbiology of the process the main challenge. Most of the microorganisms involved in the process are not identified yet; interactions between biotic (microbial dynamics) and abiotic (reactor performances) parameters are far from elucidated; the key mechanisms to target for enhancing the process are only partially known, not only in terms of bio-augmentation of key microbial populations, but also regarding organic matter degradation rates, especially hydrolysis; models and indicators to detect and prevent process failure are still being investigated, etc. In order to be able to reach the maximal potential of this technology, more comprehensive studies evaluating the process and its variation factors are therefore still needed.

Temperature and substrate composition are among the main factors affecting performance and stability of anaerobic digestion process (Ziganshin *et al*., 2013; Labatut *et al*., 2014). However, no consensus exists on the temperature range that optimizes the treatment for each substrate, since different criteria can be considered. This is a special challenge when animal manure is used, because not only the bioenergy production, but also environmental and operational aspects must be considered. Indeed, animal manure is one of the main sources of agricultural greenhouse gas emissions [concerning methane (CH_4_) and nitrous oxide (N_2_O)] (IPCC, 2006). Anaerobic digestion has been demonstrated as an effective technology for reducing CH_4_ emission from animal manure and improving the quality of manure as fertilizer (Sommer *et al*., 2004). However, the amount of carbon mineralized to biogas and used in the plants, instead of being emitted to the atmosphere, depends on the reactor conditions, mainly hydraulic retention time (HRT) and temperature. Regarding the bibliography, thermophilic conditions (> 45°C) have been reported as being superior to mesophilic conditions (25–40°C) not only in terms of reduction of pathogen load and odour emission (Sahlström, 2003; Johansen *et al*., 2013), but also due to a higher organic matter degradation rate (Nielsen and Petersen, 2000; Goberna *et al*., 2010), probably due to a more efficient exploitation of resources and a better occupation of ecological niches at higher temperatures (Goberna *et al*., 2010). However, mesophilic conditions are still recommended for treating animal manure (Labatut *et al*., 2014) based on a higher robustness of the process.

A profound comparison of thermophilic and mesophilic anaerobic digestion of cattle manure considering productive, microbiological and environmental criteria was made in the present work. For this purpose, parameters like ultimate CH_4_ yield (B_0_) in batch, CH_4_ yield in continuous reactors, potential residual CH_4_ emission, as well as physicochemical and microbial parameters were evaluated under different conditions. Three experiments were designed, where only one factor of design and operational conditions was changed in each experiment in order to optimize the robustness of the results. The factors modified were the size of the reactors (10 m^3^ versus 16 l) and the HRT (16 versus 20 days). To our knowledge, this is the first report in which microbial composition, obtained by 454 sequencing, is presented and discussed considering productive and environmental aspects in anaerobic reactors treating cattle manure under stable conditions at two different temperature regimens. The aim of the work was not only to advocate either mesophilic or thermophilic conditions for treating animal manure, but also to expand the general knowledge of the relation between physicochemical and microbial parameters in the anaerobic digestion process.

## Results and discussion

### Chemical composition

Anaerobic digestion reduced total solids (TS) and volatile solids (VS) in the manure compared with non-digested cattle manure in all experiments. As expected, samples from the thermophilic reactors showed lower content of TS and VS and higher removal VS efficiency compared with the mesophilic reactors at any period (Table 1). These facts indicate a higher organic carbon mineralization into biogas under thermophilic conditions. Digestate from thermophilic conditions showed a higher pH than that obtained from the mesophilic reactor at any period. This higher pH under thermophilic conditions has been observed previously and explained by bioenergetics balance and alkalinity differences (Labatut *et al*., 2014).

**Table 1:** Composition (average ± standard deviation) of the digestate in the reactors^a^

Parameter	Units					Pilot-scale reactors	
		Full-scale reactors		HRT = 16 days		HRT = 20 days	
		Thermophilic	Mesophilic	Thermophilic	Mesophilic	Thermophilic	Mesophilic
TS	g l^−1^	56.9 ± 1.98	58.3 ± 3.53	64.3 ± 1.65	69.7 ± 1.81	66.1 ± 1.96	70.1 ± 0.83
VS	g l^−1^	45.0 ± 2.10	47.1 ± 2.24	50.5 ± 1.45	56.3 ± 1.59	52.0 ± 1.63	56.4 ± 0.80
Removal VS efficiency	%	37.1	34.3	36.8	29.5	33.6	28.2
pH	–	7.9 ± 0.07	7.6 ± 0.07	7.9 ± 0.01	7.6 ± 0.03	8.0 ± 0.12	7.7 ± 0.07
Acetic	mg l^−1^	392.2 ± 97.24	350.9 ± 92.60	233.8 ± 14.86	199.3 ± 94.51	336.0 ± 150.74	159.0 ± 16.01
Propionic	mg l^−1^	67.8 ± 18.40	57.5 ± 21.10	87.8 ± 50.86	187.4 ± 143.26	148.0 ± 12.99	204.4 ± 193.69
Total VFA	mg l^−1^	462.8 ± 110.99	404.7 ± 130.93	321.6 ± 58.64	386.0 ± 231.07	410.0 ± 235.32	312.3 ± 177.95
Total ammonia nitrogen (TAN)	g l^−1^	2.3 ± 0.04	2.3 ± 0.13	1.4 ± 0.44	1.5 ± 0.38	2.1 ± 0.12	1.9 ± 0.15
Total kjeldahl nitrogen (TKN)	g l^−1^	2.7 ± 0.79	3.2 ± 0.85	4.2 ± 0.31	3.1 ± 0.33	3.3 ± 0.29	3.5 ± 0.16
Free ammonia	g l^−1^	0.10	0.05	0.06	0.03	0.11	0.05
Ratio TAN/TKN	%	89.1 ± 18.28	74.7 ± 13.64	42.1 ± 8.49	54.9 ± 0.65	62.1 ± 10.85	55.7 ± 1.18
Hemicellulose	% TS	11.6	11.0	8.4	13.6	12.4	11.8
Cellulose	% TS	16.5	17.7	16.7	23.2	26.4	27.0
Lignin	% TS	18.6	18.0	27.1	17.8	19.1	19.5
Lipids	% TS	3.2	3.4	2.7	2.9	2.8	2.7
Protein	% TS	13.42	13.97	12.34	10.83	10.92	10.61
B_0_	l CH_4_ kg VS^−1^	254.7 ± 17.99	266.4 ± 35.35	324.9 ± 16.69	325.3 ± 22.18	324.9 ± 16.69	325.3 ± 22.18
Methane yield	l CH_4_ kg VS^−1^	208.6 ± 27.56	154.4 ± 28.57	185.1 ± 10.55	151.2 ± 17.35	176.8 ± 10.06	155.2 ± 10.58
Residual methane	l CH_4_ kg VS^−1^	131.2 ± 5.60	171.2 ± 7.21	130.2 ± 7.40	161.4 ± 5.28	101.5 ± 6.48	140.4 ± 10.67

a Average and standard deviation calculated after 1 HRT.

Anaerobic digestion process not only considerably decreased the concentration of total volatile fatty acids (VFA) in the samples, but also changed the composition of the detected VFA pool, when comparing with the initial VFA content (Table 2). Thus, acetic and propionic acid accounted for more than 95% of total VFA measured in all reactors (Table 1); however in non-digested cattle manure, acetic and propionic acids only accounted for around 75% of the total VFA (Table 2).

**Table 2:** Composition of the cattle manures used in the three experimental periods: full-scale experiment, pilot scale working with 16 days retention time and pilot scale working with 20 days retention time

		Full-scale HRT 20 days	Pilot-scale HRT 16 days	Pilot-scale HRT 20 days
TS	g l^−1^	70.9	84.9	85.2
VS	g ^−1^	62.5	70.7	70.5
pH	–	6.47	6.4	6.7
Acetic acid	mg l^−1^	2.18	5.25	4.62
Propionic acid	mg l^−1^	5.52	2.16	1.60
Total VFA	mg l^−1^	9.65	9.59	8.35
TAN	g l^−1^	1.24	1.41	1.6
TKN	g l^−1^	2.69	3.75	3.8
Ratio TAN/TKN	%	46.1	37.6	48.2
Hemicellulose	% TS	12.2	15.3	14.8
Cellulose	% TS	16.4	20.4	11.3
Lignin	% TS	10.1	18.6	29.7
Lipids	% TS	3.8	6.4	4.1
Protein	% TS	11.6	12.3	12.0

In general, total VFA concentration was very low (lower than 500 mg l^−1^) in all reactors and periods. In addition, the observed differences in VFA concentrations between mesophilic and thermophilic reactors were in general low in all experiments. However, some slight differences on individual VFA were observed. Total VFA and acetic acid were observed to be higher in thermophilic conditions than in the mesophilic reactors in both scales working with 20 days HRT. On this regard, Kim and colleagues (2002) found significant higher VFA concentration under thermophilic conditions compared with the mesophilic range, especially in terms of total VFA and acetic acid concentrations. In contrast, higher total VFA and propionic acid concentrations were found in the mesophilic lab-scale reactor working at 16 days HRT compared with its corresponding thermophilic reactor.

As expected, total ammonia nitrogen (TAN) concentration and the ratio TAN/total Kjeldahl nitrogen (TKN) increases in all reactors after the anaerobic digestion, as a result of the urea and protein hydrolysis (Sung and Liu, 2003). However, no clear differences were observed in TAN, or in TKN, between temperature ranges in any period. These results contrast with literature where higher TAN concentrations were related under thermophilic conditions in reactors fed with animal manure (Labatut *et al*., 2014). Concerning the calculated free ammonia concentration (NH_3f_), doubled NH_3f_ was obtained in thermophilic reactors compared with mesophilic reactors in all periods (Table 1), probably due to the higher temperature and pH in thermophilic reactors. However, NH_3f_ concentration was lower than the reported as inhibitory in all reactors (Hansen *et al*., 1998).

Differences in the organic fractions between thermophilic and mesophilic reactors were not evident in any of the experiments working with 20 days HRT (Table 1). Similarly, there were only slight differences in lipid and protein concentrations under thermophilic and mesophilic conditions at 16 days HRT. However, higher hemicellulose and cellulose degradation was obtained under thermophilic than under mesophilic conditions in the lab-scale reactors working with 16 days HRT. This clearly demonstrates that fibers are among the slowest degradable organic compounds in the reactors, and that the mesophilic microbial community working at 16 days HRT is only able to degrade the fiber fraction of the input material inefficiently. Hence, to ensure efficient degradation of the less-degradable fiber fraction in cattle manures, reactors operated at 16 days HRT should preferably run under thermophilic conditions, while mesophilic conditions take longer HRT (demonstrated here with 20 days HRT) to obtain the same degradation efficiency.

### Methane yield

The B_0_ determination of the manure in batch assay, showed that manure used in the lab-scale experiment have a higher B_0_ value than manure used in the pilot-scale reactors (Table 1). However, no differences were found when comparing B_0_ determined under mesophilic and thermophilic conditions in each scale. Our results are in agreement with Hashimoto and colleagues (1981) and Palatsi and colleagues (2009), who tested a range of temperatures on cattle manure and a mixture of secondary sludge and municipal wastewater treatment plant, respectively, without finding significant differences on B_0_ between thermophilic and mesophilic conditions.

**Figure 1:**
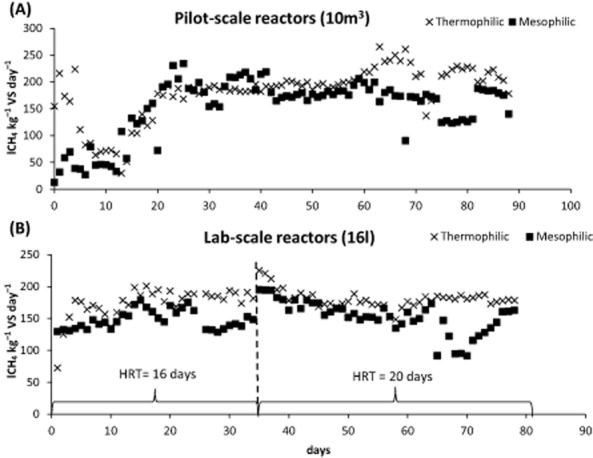
Methane yield (lCH_4_ kg^−1^ VS day^−1^) obtained in the pilot-scale experiment (A) and in the lab-scale experiment (B). In the lab-scale experiment, two HRT were used, and the corresponding periods are marked in the Fig. 1B.

Methane yield in the pilot-scale and the lab-scale experiments was registered continuously throughout the experiments (Fig. 1). However, due to technical problems with stabilizing the temperature in the mesophilic lab-scale reactor at day 65, CH_4_ yield data between day 65 and day 75 are not included in our calculations.

**Figure 2:**
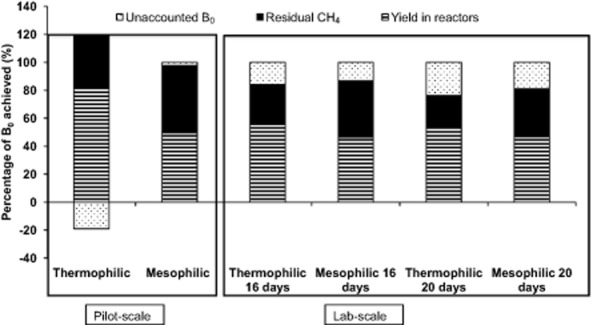
Percentage of B_0_ in terms of l CH_4_ l slurry^−1^ achieved in the reactors and in the subsequent batch to determine residual gas. The difference between the sum of the CH_4_ yield retrieved in reactors and in the residual batch and the B_0_ is considered as unaccounted gas_._

Figure 2 shows the percentage of B_0_ achieved in the reactors, in the residual batch and the unaccounted amount of CH_4_ when comparing with B_0_ determined in batch assay. In general, more than 40% of B_0_ was retrieved in continuous reactors in all experiments. Concerning differences between temperatures, thermophilic reactors showed higher CH_4_ yield than the respective mesophilic reactors, not only in terms of lCH_4_ kg VS^−1^ (Table 1), but also in terms of percentage of B_0_ achieved, especially in the pilot-scale reactors. In pilot-scale reactors, average CH_4_ yield after stabilization was 208.6 l CH_4_ kg VS^−1^ under thermophilic conditions resulting in retrieval of 80% of the B_0_ (Fig. 2) and 154.4 l CH_4_ kg VS^−1^ under mesophilic conditions, retrieving less than 50% of the B_0_ (Fig. 2). This means that more than 35% extra CH_4_ is retrieved under thermophilic conditions compared with the mesophilic range in the pilot-scale reactors. This complies with the study carried by Mackie and Bryant (1995), which states that a higher CH_4_ yield and energetic energy input (kJ day^−1^) can be achieved in reactors working at thermophilic range (60°C) compared with mesophilic (40°C) range. The higher VS reduction and increased CH_4_ yield obtained under thermophilic conditions show that, even though the theoretical CH_4_ yield (B_0_) is similar regardless of temperature, a higher amount of VS can be transformed into CH_4_ if temperature is high compared with low temperature when hydraulic retention time is equally limiting.

Differences between temperature ranges on CH_4_ yield were less apparent in the lab-scale reactors (Table 1), presumably due to the effect of scale on reactor performance, but little has been found in the literature on this regard. Differences between scales could also be due to the fact that the slurry used in each scale was collected at different periods.

In the lab-scale reactor, working with 16 days HRT, increasing temperature from mesophilic to thermophilic range resulted in a 22% increased CH_4_ yield. However, the difference on CH_4_ yield between temperatures ranges was damped at higher HRT; in fact, only an improvement of 14% in CH_4_ production was observed, when comparing thermophilic and mesophilic conditions in lab-scale reactors whit 20 days HRT. Therefore, mesophilic conditions at 16 HRT could result in lower VS degradation compared with either higher HRT or increased temperature; and thus, increasing HRT mainly affects CH_4_ yield at mesophilic conditions in contrast to thermophilic conditions, where CH_4_ yield remains more constant. According to these results, one could conclude that increasing the HRT from 16 to 20 days could not lead to improvements in the CH_4_ yield at thermophilic conditions. However, residual CH_4_ production decreased around 22%, when increasing the HRT from 16 to 20 days under thermophilic conditions (Table 1). This means that less VS is left un-degraded after anaerobic digestion under thermophilic conditions at 20 days compared with 16 days HRT. The reason why this high VS degradation was not associated with an increased CH_4_ yield in the lab-scale reactor at 20 days HRT under thermophilic conditions is unclear, but errors of sampling and analysis might be involved. This could also explain the differences in the effect of temperature between the two reactor scales.

Around 20% of decrease in residual CH_4_ yield was reached under thermophilic conditions compared with the corresponding mesophilic conditions in any period (Table 1). This indicates that the resulting digestate from the mesophilic digestion contained a higher amount of undigested biodegradable VS, which potentially can be degraded during storage resulting in considerable loss of CH_4_ to the atmosphere. In contrast, digestion under thermophilic conditions results in a higher biogas production and reduced potential for CH_4_ emissions to the atmosphere, when the retention time is the same. Hence, the production of biogas under thermophilic conditions may not only positively impact the economical revenue for farmers, but in addition also reduce the environmental fingerprint of manure management. However, increased costs for heating and installation of thermophilic digesters should be taken into account if a total economic and environmental balance should be made.

In pilot-scale reactors, the percentage of unaccounted B_0_ was very low under mesophilic conditions (Fig. 2) and negative under thermophilic conditions. In the lab-scale reactors, although more than 75% of B_0_ was accounted in the lab scale in all periods, the percentage of unaccounted B_0_ was higher than in the pilot scale, especially working with 20 days HRT. This could be explained by the low representativeness of the slurry sampled for B_0_ analysis. In fact, slurry used for B_0_ analysis was sampled only once in both experiments at the end in the pilot-scale reactors experiment (after 2 months storage) and at the beginning in the lab-scale reactors experiment (fresh manure). Losses of easy degradable VS, during manure pre-storage in the lab-scale experiments could explain the higher fraction of unaccounted B_0_. Methane production in reactors and sampling after the continuous process for residual gas production determination were done in the last days of the experiment, comparing these values with B_0_ from the fresh manure might explain the deviations.

Differences in the B_0_ yield between scales might also be explained by the fact that fresh manure was used in lab-scale batch, while stored manure was used in the batch from pilot scale.

### Microbial community composition

**Figure 3:**
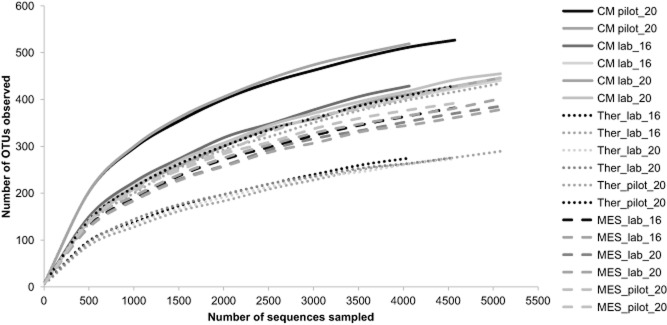
Species accumulation (rarefaction) curves for all samples investigated (nine samples per duplicate), being: the three cattle manures used to feed the reactors in the three experimental periods: pilot-scale run with 20 days HRT (pilot_20), lab-scale run with 16 days HRT (lab_16) and lab-scale run with 20 days HRT (lab_20); and the reactor samples in each experiment obtained from the thermophilic (Ther) and the mesophilic (MES) reactors.

All sequenced samples, which were deposited at Sequence Read Archive (SRA) with accession number PRJNA263043, showed similar levels of observed operational taxonomic units (OTUs; Table 3). In addition, the replicates were similar, both in terms of observed OTUs and in terms of predicted diversity (Chao 1 and Shannon indices), indicating a good similarity between replicates. The rarefaction curves (Fig. 3) reached clear asymptotes for all samples indicating that the number of sequences per sample was high enough to cover the microbial diversity in each sample. The highest genotypic diversity was observed in both cattle manures as compared with the reactors digestate; especially in the cattle manure used in the full-scale experiment. The lowest number of OTUs and the lowest diversity index were observed under thermophilic conditions in all experimental periods, followed by mesophilic conditions. This is in good accordance with Sekiguchi and colleagues (1998), likewise observing lower cumulative sequence numbers under thermophilic conditions (55°C) compared with mesophilic conditions (35°C) in up-flow anaerobic sludge blanket reactors working with artificial waste water.

**Table 3:** Sequencing sample overview and estimated diversity

Sample	Experiment	HRT	Cleaned reads	Observed number of OTUs	Estimated diversity		Average		
							Observed number of OTUs	Estimated diversity	
					Chao1	Shannon indices		Chao1	Shannon indices
Thermophilic	Pilot scale	20	5,949	434	599	6.19	432	607	6.21
Thermophilic	Pilot scale	20	4,667	429	614	6.23			
Mesophilic	Pilot scale	20	6,227	447	647	6.10	420	588	6.21
Mesophilic	Pilot scale	20	4,984	392	529	6.32			
Cattle manure	Pilot scale	20	4,733	527	626	7.55	523	623	7.58
Cattle manure	Pilot scale	20	4,239	519	620	7.61			
Cattle manure	Lab scale	16	4,246	429	608	6.10	435	591	6.03
Cattle manure	Lab scale	16	5,360	441	573	5.95			
Thermophilic	Lab scale	16	4,559	275	421	4.92	283	427	4.87
Thermophilic	Lab scale	16	5,658	290	432	4.82			
Mesophilic	Lab scale	16	4,603	382	528	6.10	392	525	6.13
Mesophilic	Lab scale	16	5,846	402	522	6.16			
Cattle manure	Lab scale	20	5,183	446	600	6.01	451	605	6.03
Cattle manure	Lab scale	20	5,721	455	610	6.05			
Thermophilic	Lab scale	20	4,653	274	359	4.93	275	351	4.99
Thermophilic	Lab scale	20	4,739	276	343	5.04			
Mesophilic	Lab scale	20	5,811	386	536	6.16	382	551	6.14
Mesophilic	Lab scale	20	6,205	378	566	6.11			
Total number of raw reads		111 662	–	–	–	–	–	–	–
Total number of cleaned reads		93 383	–	–	–	–	–	–	–

**Figure 4:**
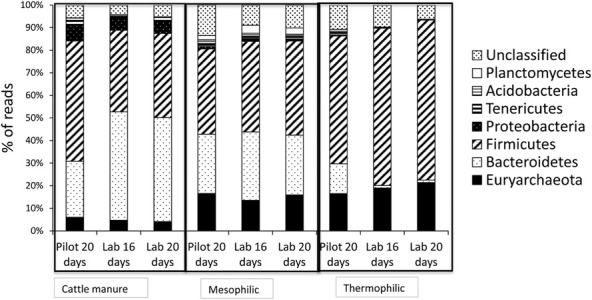
Microbial community composition as percentage of total reads at phylum level in the cattle manures used to feed the reactors and in the thermophilic (50°C) and the mesophilic (35°C) reactors at the end of each experimental period. Each measure represents the average of two replicates.

Generally, bacterial communities were dominated by Firmicutes and Bacteriodetes. These two phyla represented close to 80% of the total numbers of the reads obtained from cattle manure fed to the reactors (Fig. 4). *Bacterioidetes* and Firmicutes also represented the dominating phyla in both groups of reactors (around 70% of the total reads in both groups of reactors) although lower in relative abundance in the reactors compared with the cattle manure. This was likely due to an increase in relative abundance of Euryarchaeota in the reactors, especially under thermophilic conditions. In fact, Euryarchaeota in the cattle manure only represented around 5% of the total reads, but around 15% in all the reactors. Bacterioidetes and Firmicutes have been pointed out as being dominant with respect to numbers of both sequences and species-level detected in rumen (Kim *et al*., 2011), in human gut (Karlsson *et al*., 2011) and in reactors working with different organic compounds (Klocke *et al*., 2007; Kröber *et al*., 2009; Kampmann *et al*., 2012). *Proteobacteria* was abundant only in cattle manure (around 6% of the total reads), while markedly reduced in mesophilic reactors (around 1% of the total reads) and hardly detectable in the thermophilic reactors (Fig. 4).

Concerning differences in bacterial community composition between temperature ranges, bacterial diversity decreased towards a clear Firmicutes dominance under thermophilic conditions in all experimental periods. This is in good accordance with other observations of Firmicutes as the dominating phylum during thermophilic digestion of municipal (Tang *et al*., 2004) and household waste waters (Levén *et al*., 2007).

Cattle manure used in the two lab-scale experiments showed similar bacterial community composition, probably because the only difference between these two manures was the storage time. However, the manure used in the pilot-scale experiment showed a lower percentage of Bacteriodetes reads and higher percentage of Firmicutes reads compared with that used in lab-scale experiment. These differences in bacterial composition among manures were hardly reproduced on reactors. In fact, mesophilic reactors showed a similar bacterial composition in all periods (around 40% of the total reads belonged to Firmicutes and 30% of the total reads belonged to Bacteroidetes). In the thermophilic full-scale reactors, Bacteriodetes represented 13% of the total reads and Firmicutes 60%. In pilot scale reactors however, Bacteriodetes were hardly detected (less than 2% of the total reads) and Firmicutes represented around 70% of the total reads. These results clearly show that temperature had a higher impact on microbial composition in the reactors than initial microbial composition of the manure or HRT. These results contrast with previous works in which the original microbial composition of the animal slurry has been identified as an important factor in determining microbial composition of the anaerobic digesters (Moset *et al*., 2014a).

**Figure 5:**
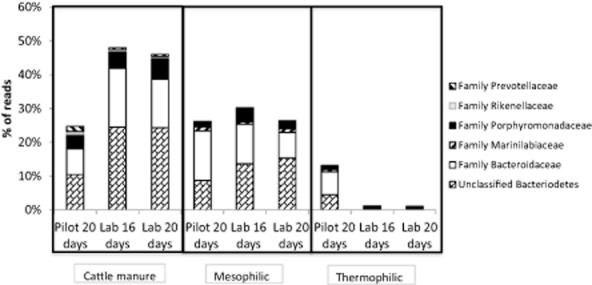
Phylum *Bacterioidetes* community composition as percentage of reads at family level in the cattle manures used to feed the reactors and in the thermophilic (50°C) and the mesophilic (35°C) reactors at the end of each experimental period. Each measure represents the average of two replicates.

In cattle manure and mesophilic reactors, Bacteroidaceae and Porphyromonadaceae were the dominant families of the phylum Bacteriodetes (Fig. 5). These two families represented around 20% of the total reads in the cattle manure used to feed the reactors and around 15% of the total reads in the mesophilic reactors. The genus *Bacteroides* belonging to the Bacteroidaceae family is the most abundant core component of the human gut microbiota (Karlsson *et al*., 2011), where they are considered as carbohydrate-utilizing bacteria (Klocke *et al*., 2007). In addition, members of the Bacteroidaceae family have been characterized as important cellulolytic microorganism (Khan *et al*., 1980; Murray, 1986).

Within the Phylum Firmicutes, members of the Clostridia class were more abundant than members of the Bacilli class in all groups of reactors and in cattle manure. The percentage of reads belonging to the Clostridia class was higher in thermophilic than in mesophilic reactors and in the cattle manures, especially in the pilot-scale thermophilic reactor, where the Clostridia class represented more than 60% of the total reads. Levén and colleagues (2007) also observed a higher abundance of reads associated with Clostridia in thermophilic (55°C) household waste reactors compared with mesophilic (37°C) counterparts. These authors attributed this fact to a heat activation of the spore-forming Clostridia under thermophilic conditions. Additionally, some homoacetogenic Clostridia may be responsible for the first step in syntrophic acetate oxidation to CH_4_ (Walter *et al*., 2012). These homoacetogens may perform both directions depending on concentrations of substrates and products in the reactor, the acetate oxidation to CO_2_ and H_2_, or the acetate synthesis from CO_2_ and H_2_ (Schink, 1997) that is subsequently converted to CH_4_ by hydrogenotrophic methanogens. This syntrophic relationship has been found favoured under thermophilic conditions (Schink, 1997; Qu *et al*., 2009; De Vrieze *et al*., 2012; Walter *et al*., 2012; Ho *et al*., 2013).

**Figure 6:**
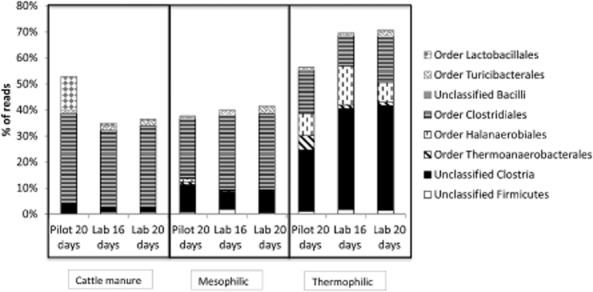
Phylum Firmicutes community composition as percentage of reads at order level in the cattle manures used to feed the reactors and in the thermophilic (50°C) and the mesophilic (35°C) reactors at the end of each experimental period. Each measure represents the average of two replicates.

Within the Clostridia class, members of the *Clostridiales* order dominated in the cattle manures (around 30% of the total reads) and in the mesophilic reactors (more than 20% of the total reads) (Fig. 6). The thermophilic reactors, however, were dominated not only by members of the *Clostridiales* order (around 15% of the total reads), but also by members of the Halanaerobiales order (around 10% of the total reads) especially members of the Halanaerobiaceae family and other unknown Clostridia (around 20% of the total reads in full-scale thermophilic reactor and around 40% of the total reads in pilot-scale thermophilic reactors).

**Figure 7:**
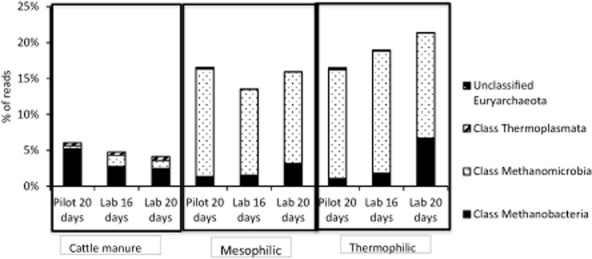
Phylum *Euryachaeota* community composition as percentage of reads at class level in the cattle manures used to feed the reactors and in the thermophilic (50°C) and the mesophilic (35°C) reactors at the end of each experimental period. Each measure represents the average of two replicates.

**Figure 8:**
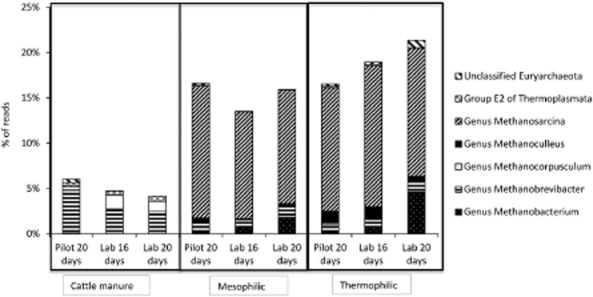
Phylum *Euryachaeota* community composition as percentage of reads at genus level in the cattle manures used to feed the reactors and in the thermophilic (50°C) and the mesophilic (35°C) reactors at the end of each experimental period. Each measure represents the average of two replicates.

In all samples, the three dominant classes of Euryarchaeota were Methanomicrobia, Methanobacteria and Thermoplasmata (Fig. 7). There were marked differences between reactors and cattle manures, not only in terms of relative abundance of Euryarchaeota that, as previously stated, was higher in the reactors than in the manures, but also through the dominance of Methanomicrobia class in the reactors, whereas Methanobacteria class dominated in the cattle manures (Fig. 7). In addition, members of the Thermoplasmata class (Group E2; Lino *et al*., 2013) (Fig. 8) were detected in the cattle manures, but hardly so in the reactors. In cattle manures, the major part of Euryarchaeota reads (Fig. 8) belonged to the genera *Methanobrevibacter* (order Methanobacteriales) and *Methanocorpusculum* (order Methanomicrobiales), in which all members are hydrogenotrophs (H_2_/CO_2_-utilizing methanogens) (Angelidaki *et al*., 2011). Species of *Methanobrevibacter* have been found to dominate in the rumen due to their high growth rate and their ability to competitively utilize H_2_ and CO_2_ (Kim *et al*., 2011). *Methanobrevibacter* species have also been isolated from the intestinal tracks of animals and humans, as well as from animal manure (Garcia *et al*., 2000). Whitehead and Cotta (1999) and Yamamoto and colleagues (2011) detected *Methanocorpusculum* in swine waste storage pits and in cattle manure compost during the first days of composting.

However, this *Euryachaeota* genus was hardly detected in any of the reactors. In fact, our results clearly show that while methanogens retrieving energy through hydrogenotrophic methanogenesis dominate in the manures, potential representatives of the other physiological groups of methanogens thrive when the manure is further digested in the reactors, regardless of temperature. In fact, the dominating Euryachaeota genera in the reactors were *Methanosarcina* (order Methanosarcinales; family Methanosarcinacea*e*), with more than 12% of the total reads, and *Methanobacterium* (order Methanobacteriales), with around 2% of total reads (Fig. 8). Contradictory results can be found in bibliography regarding methanogenic dominance in anaerobic reactors. Nettmann and colleagues (2010), Song and colleagues (2010) and Moset and colleagues (2014b) reported Methanobacteriales and Methanomicrobiales as the dominant methanogenic orders in anaerobic reactors working with animal manure. However, Karakashev and colleagues (2005) studied methanogenic community variations in 15 Danish full-scale biogas plants running on different substrates, temperatures and HRT, and found Methanosarcinaceae to be the dominating family in most manure reactors. Walter and colleagues (2012) found *Methanosarcina* species in reactors where syntrophic acetate oxidation was the dominant CH_4_ production pathway.

From an energetic point of view, acetoclastic methanogenesis should be more favourable in anaerobic reactors than hydrogenotrophic methanogenesis (De Vrieze *et al*., 2012). However, hydrogenotrophic methanogenesis has been pointed as more favourable under some conditions like changes in substrate composition (Moset *et al*., 2014b), high VFA concentration in reactors (Hori *et al*., 2006) or as stated above, thermophilic conditions (Schink, 1997). Dominance of *Methanosarcina* could be explained by the fact that members of this genus can use both the acetoclastic and the hydrogenotrophic pathway (De Vrieze *et al*., 2012). Therefore, as stated by De Vrieze and colleagues (2012), the establishment of the syntrophy between homoacetogenic bacterium (as members of Clostridia class) and *Methanosarcina* members could increase the stability of the process in anaerobic reactors at both, mesophilic and thermophilic conditions.

## Experimental procedures

### Substrate and reactors performances

Dairy cattle manure was obtained from Research Centre Foulum (Aarhus University, Denmark) in two periods (April and June). The cattle manure sampled in April was used to feed the pilot-scale reactors; the manure sampled in June was used in the two lab-scale reactors. Composition of the cattle manures used was characterized prior to the start the three experimental periods (Table 2). Mesophilic and thermophilic inoculums were obtained from a mesophilic and thermophilic reactor, respectively, located at Research Centre Foulum (Aarhus University, Denmark). Both reactors had been running for more than 1 year under these temperature conditions.

Before starting up the experiments, reactors used in each experiment were completely filled with their corresponding inoculum.

#### Pilot-scale reactors

The pilot-scale experiment was run for 90 days (more than three times HRT) in two continuously stirred tank reactors (10 m^3^). The two reactors were similar in design, i.e. constructed in stainless steel and heated by an external water jacket. One reactor was operated under thermophilic conditions (50°C) and one under mesophilic conditions (35°C). Continuous mixing of the digestate in the reactors was obtained using a central shaft with a propeller at the bottom, rotating at 60 r.p.m. Gas production was measured with a differential pressure transmitter device (EJX110A Yokogawa, Japan). Feeding and unloading the reactors was performed automatically by electric pumps, and the exact amount (500 kg day^−1^) of manure fed and unloaded was controlled by weighing in order to obtain an HRT of 20 days, achieving a constant organic loading rate of 3.1 g VS l^−1^ day^−1^.

#### Lab-scale reactors

The lab-scale experiment was run for 78 days in total using two continuously stirred tank reactors with 20 l total capacity and 16 l working capacity. The experimental period was divided into two in which two different HRT were used; during the first 34 days, the reactors were working at 16 days HRT, and during the last 44 days, the reactors were working at 20 days HRT.

In the two lab-scale reactors, mixing was performed by a central shaft with two propellers one at the bottom and one in the middle, continuously rotating at 60 r.p.m. The reactors were heated by electrical resistances at the bottom, and the tank temperature was controlled by a temperature probe. The reactors were manually fed and unloaded daily with the amounts needed to obtain HRTs of 16 or 20 days and 4.4 g VS l^−1^ day^−1^ or 3.5 g VS l^−1^ day^−1^ respectively. Gas production in this case was measured using an automatic CH_4_ potential system (AMPTS II, Bioprocess Control, Beijing, China).

### Ultimate methane yield and residual digestate methane production

Ultimate methane yield as the maximal CH_4_ production of each cattle manure used (pilot-scale and lab-scale experiments) in terms of l CH_4_ kg^−1^ of VS was determined in a batch assay. The batch assay was added the same manure as used to feed the reactors and with inoculum extracted from the running reactors. Prior to running the batch assay, the inoculums were pre-incubated for 15 days at their corresponding temperatures (mesophilic or thermophilic) in order to deplete the residual biodegradable organic material (degasification) as recommended by Angelidaki and colleagues (2009). Six bottles per manure were filled with 150 ml of inoculum and the corresponding cattle manure to maintain an inoculum-manure ratio of approximately 1:1, determined on VS basis. Inoculum: substrates ratio higher than 0.7 on VS basis has been previously tested with slow biodegradable substrates with successful results (Hashimoto, 1989; Møller *et al*., 2004; Raposo *et al*., 2009).

After filling, each bottle was sealed with a butyl rubber stopper and aluminium crimps, and the headspace was flushed with pure N_2_ for two minutes. Three bottles per manure were then incubated at thermophilic (50°C) and three bottles at mesophilic conditions (35°C) for 100 days.

Digestate samples from mesophilic and thermophilic reactors were also taken at the end of each experimental period to determine the residual CH_4_ production. Three bottles (0.5 l) per reactor in each experimental period were prepared and filled with 200 mg of the digestate obtained. After filling, each bottle was sealed with a butyl rubber stopper and aluminium crimps, and the headspace was flushed with pure N_2_ for 2 min.

The measurement of biogas production in bottles from both batch experiments was done by inserting a needle, connected to a tube with inlet to a column filled with acidified water (pH < 2), through the butyl rubber. The biogas produced in each bottle was calculated by the water displaced until the two pressures (column and headspace in bottles) were equal. The volume of biogas produced was measured immediately after bottles were taken out from the incubators at the two temperature ranges. The CH_4_ produced in this study is expressed at standard conditions for temperature and pressure (STP, temperature = 273.15 K, pressure = 100 kPa) according to the recommendations made by Angelidaki and Sanders (2004).

### Analyses

#### Physicochemical composition

Digestate samples from each reactor were taken weekly and analysed for pH, dry matter (as a TS content) and organic matter (as VS content), following the TS and VS procedure (APHA, 2005) respectively. Dissolved VFA were determined weekly using a gas chromatograph (5560-D of APHA, 2005) equipped with a flame ionization detector (HP 68050 series Hewlett Packard). Total ammonia nitrogen was also determined weekly from fresh samples using photometric kits (Spectroquant kit, Merk, USA).

At the end of each experimental period, samples from each reactor were taken, dried (48 h at 60°C) and milled using a mill with a 0.8 mm of diameter (Cyclotec 1093, Foss, North America). Fiber fractions (total neutral detergent fiber, acid detergent fiber and lignin), crude fat and TKN were analysed from the dried milled samples. Fiber fractions were determined according to the Van Soest procedure (Van Soest, 1991) and corrected for ash. Crude fat was determined by measuring extracted lipids with petroleum ether (Soxtec 2050, Foss analytical, Hillerød, Denmark) after hydrolysing with HCl (Stoldt, 1952). Total kjeldahl nitrogen was determined according with APHA (2005).

#### Biogas composition

Biogas samples were taken from reactors twice a week and from bottles in each biogas measurement. In all cases, biogas was sampled by flushing a 22 ml sample bottle with 300 ml of biogas. Biogas composition, in terms of carbon dioxide, CH_4_ and hydrogen sulfide concentration, was determined using a gas chromatograph equipped with a thermal conductivity detector (Agilent technologies 7890A).

#### Microbial community composition by 454 sequencing

Samples from each reactor and from the manure were taken at the end of each experiment to determine microbial community composition (nine samples) by 454 sequencing. Total nucleic acids were extracted from 300 μg of sample (per duplicate) after thawing using E.Z.N.A. Stool DNA Kit (OMEGA, Bio-Tek). The DNA concentrations were determined using a NanoDrop ND-1000 spectrophotometer (Thermo Fisher Scientific, Wilmington, DE).

Amplification of the extracted DNA was carried out on a DOPPIO thermal cycler (VWR, Radnor, Pennsylvania, USA). Fusion primers for 454 FLX Titanium sequencing were constructed based on combined bacterial and archaeal-targeting primers ArBa515F and ArBa806R (Kittelmann *et al*., 2013). Eighteen unique 10-base long barcode regions were included in the forward primers to allow mixing and sequencing of samples in one pool and subsequent assignment of reads to individual samples. The polymerase chain reaction (PCR) was performed in a final volume of 50 μl containing 1 μl of template DNA, 2 μl of dNTP (2 mM of each nucleotide μl^−1^), 5 μl of DyNazyme buffer 10X, 2 μl of each primer (5 pmol μl^−1^), 0.5 μl of DyNazyme II polymerase (2 U μl^−1^) and 37.5 μl of ddH_2_O. The cycling conditions were denaturation at 95°C for 3.5 min, followed by 30 cycles of 92°C for 30 s, 52°C for 30 s and 72°C for 30 s, and finally an extension step at 72°C for 10 min. Polymerase chain reaction products were visualized in 2% agarose gel stained with ethidium bromide and purified using Qiaquick purification kit (Qiagen, Germany). Purified PCR products were quantified on the NanoDrop ND-1000 spectrophotometer, and equal amounts of product from each sample were mixed and sequenced at MWG Eurofins (Braunsweig, Germany).

Sequence analysis was done using Qiime pipeline version 1.7.0 (Caporaso *et al*., 2010a). Reads were de-noised (Reeder and Knight, 2010), sorted between samples, and primers were removed giving trimmed sequences of on average 273 bp (minimum 123 bp and maximum 314 bp). Operational taxonomic units were picked de novo from quality-filtered reads using a 97% similarity cut off (Edgar, 2010). Reads were aligned using PyNAST (Caporaso *et al*., 2010b) against the Greengenes Core reference alignment (DeSantis *et al*., 2006), and unaligned reads were removed. Chimeras were detected using uchime (Edgar *et al*., 2011) using usearch (Edgar, 2010). Chimeras, as well as singleton sequences, were removed. Taxonomy was assigned to representative sequences for each OTU using rdp Classifier 2.2 (Wang *et al*., 2012) based on the Greengenes taxonomy and references database (v. 12.10) (McDonald *et al*., 2012; Werner *et al*., 2012). Alpha diversity and rarefaction curve for each sample was calculated.

### Calculations

The percentage of VS degraded from the manure during the anaerobic digestion process (VS removal efficiency) was calculated as a mass balance from VS content in the slurry and in the reactors taking VFA losses during the drying process into account, as shown in Moset and colleagues (2012). Dissolved VFA content in the manure was added to VS fraction when calculating the VS removal efficiency in the following manner: 100% of VFA was added to VS in manures with a pH below 7, 80% of VFA was added to VS in manures with pH between 7 and 8, 10% of VFA was added to VS in manures with a pH higher than 8.

Total free ammonia in the digestate was calculated in each experiment using the equations described by Budavari (2001) as a function of the temperature, pH and TAN as shown in eqn 1:

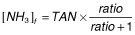
1

Where [NH_3_]_f_ is free ammonia concentration, TAN is the average TAN measured in each reactor, and ratio is the ratio of distribution between ammonia and ammonium in the manure. This ratio depends on manure’s pH and temperature manure as shown in eqn 2:

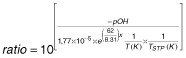
2

Where T is the temperature in the sludge (Kelvin), and T_STP_ is the temperature in standard conditions.

In each period, average digestate composition and CH_4_ yield were determined after one HRT. Methane yield in reactors, residual CH_4_ production and B_0_ were determined in terms of l CH_4_ per l manure added to determine the proportion of B_0_ reached for the complete system with a continuous process combined with a post-digestion until only a negligible amount of gas was produced. The sum of the gas in the complete system should in theory be equal to B_0_ of the manure since both methods measure the gas production until the gas production almost cease. Therefore, deviations would be expected to be caused by analytical uncertainty, sampling errors, etc. The deviations in the gas yield determined by the two methods will be considered as unaccounted B_0_.

## Conclusions

The anaerobic digestion process was achieved at both temperatures tested. Not only because CH_4_ was produced in all reactors, but also because chemical and microbial composition in reactor contents showed some similarities, independent of temperature conditions, when compared with undigested animal manure. These indicators of the anaerobic digestion process were mainly in terms of decrease is TS, VS and VFA concentration and increase in pH, TAN and Euryarchaeota and *Bacteria* intensification towards *Methanosarcina* genus and Clostridia class members respectively. In addition, no differences in B_0_ were found between temperatures conditions in any manure used.

According to the results obtained in this study and taking into account the indicators listed above, we can conclude that thermophilic conditions completed the anaerobic digestion degradation of organic matter more successfully. In fact, under thermophilic conditions, a higher CH_4_ yield and percentage of B_0_ and lower residual CH_4_ emission were retrieved. Therefore, thermophilic conditions are recommended, especially when working with high-fiber organic substrates at HRT lower than 20 days.

The results obtained in this work also showed that temperature had higher impact on reactor performance than reactor size (10 m^3^ versus 16 l), or HRT (16 versus 20 days).

## Conflict of Interest

None declared.
